# Phillyrin Attenuates Osteoclast Formation and Function and Prevents LPS-Induced Osteolysis in Mice

**DOI:** 10.3389/fphar.2019.01188

**Published:** 2019-10-17

**Authors:** Jing Wang, Gang Chen, Qianqian Zhang, Fuli Zhao, Xiaolu Yu, Xuemei Ma, Mei Liu

**Affiliations:** Jiangsu Key Laboratory for Molecular and Medical Biotechnology, College of Life Sciences, Nanjing Normal University, Nanjing, China

**Keywords:** phillyrin, osteoclast formation, bone resorption, MAPK signaling pathway, osteolysis

## Abstract

As the sole cell type responsible for bone resorption, osteoclasts play a pivotal role in a variety of lytic bone diseases. Suppression of osteoclast formation and activation has been proposed as an effective protective therapy for new bone. In this study, we reported for the first time that phillyrin (Phil), an active ingredient extracted from forsythia, significantly inhibited RANKL-induced osteoclastogenesis and bone resorption *in vitro* and protected against lipopolysaccharide-induced osteolysis *in vivo*. Further molecular investigations demonstrated that Phil effectively blocked RANKL-induced activations of c-Jun N-terminal kinase and extracellular signal-regulated kinase, which suppressed the expression of c-Fos and nuclear factor of activated T-cells cytoplasmic 1. Taken together, these data suggested that Phil might be a potential antiosteoclastogenesis agent for treating osteoclast-related bone lytic diseases.

## Introduction

Bone metabolism is a continuous and dynamic remodeling process that is maintained by bone formation and bone resorption. Bone resorption is executed by osteoclasts, which differentiates from the hematopoietic mononuclear precursors upon stimulation by macrophage colony-stimulating factor (M-CSF) and receptor activator of nuclear factor κB ligand (RANKL). M-CSF is essential for survival and proliferation of osteoclast precursors, while RANKL is an indispensable cytokine for osteoclast differentiation and activation. RANKL interacts with its receptor RANK to recruit tumor necrosis factor (TNF) receptor–associated factor 6 and further activate a cascade of intracellular signaling pathways such as nuclear factor κB (NF-κB), phosphatidylinositol 3 kinase/protein kinase B, and mitogen-activated protein kinases (MAPKs) (Boyle et al., 2003; Kaji et al., 2010; Rosen et al., 2013). Activations of these pathways promote the expression and activation of nuclear factor of activated T-cells cytoplasmic 1 (NFATc1), which triggers the transcript of osteoclast-specific genes and ultimately results in the formation of multinucleated osteoclasts (Asagiri et al., 2005; Masataka and Hiroshi, 2007; Iezaki et al., 2016). As the sole cell type responsible for bone resorption, osteoclasts play a critical role in maintaining normal bone structure. Enhanced osteoclast formation and activation can lead to a variety of osteolytic bone diseases such as osteoporosis, aseptic loosening of prostheses, Paget disease of bone, and erosive arthritis (Lerner, 2006; Greenfield et al., 2010; Reddy, 2010). Thus, the osteoclast has become an important target for the treatment and prevention of osteopathic diseases.

**Figure 1:**
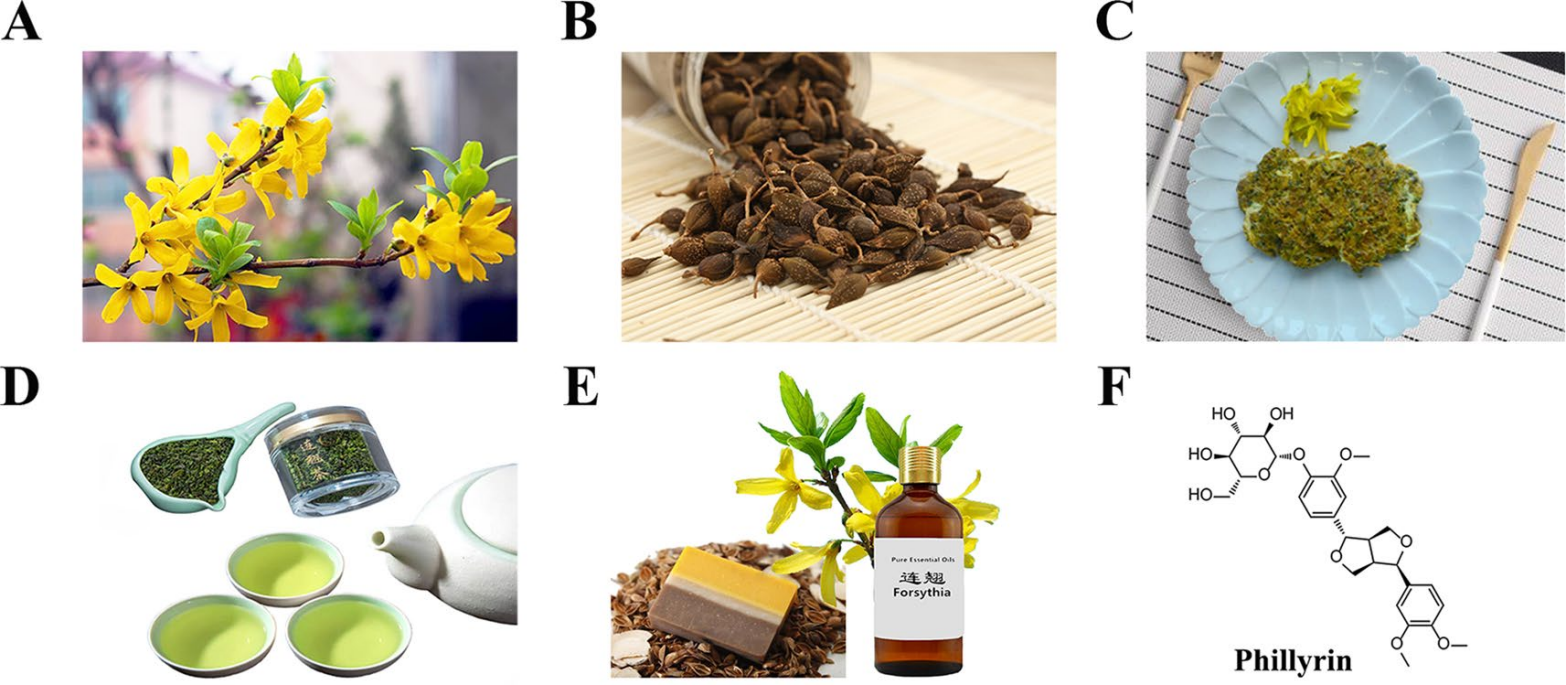
*Forsythia suspense* and Phillyrin (Phil). **(A)** The flowers and leaves of *Forsythia suspense*. **(B)** The fruits of *Forsythia suspense*, namely, “Lianqiao” in Chinese. **(C)** The flowers can be used to make forsythia flower-egg pancakes. **(D)** The leaves can be used to make forsythia-tea. **(E)** The extracts from *Forsythia suspense* have often been used as an ingredient in the cosmetic industries (such as forsythia oil and forsythia soap). **(F)** The molecular structure of Phil.

*Forsythia suspensa* (Thunb.) Vahl (Oleaceae) is a very important herbal medicine and has been widely used in clinic to treat various infectious diseases (Kuang et al., 1988; Kuo et al., 2014; Wang et al., 2018). Moreover, in our daily life, *F. suspensa* and its extracts have often been found as an ingredient in the food, beverage, and cosmetic industries (Yang et al., 2004; Kim et al., 2006) (**Figures 1A–E**). Phillyrin (Phil), one of the main natural lignans extracted from *F. suspensa*, has been reported to possess a wide range of pharmacological properties, including antioxidation, antivirus, anti-inflammation, antiobesity, and antipyretic activity (Qu et al., 2010; Liu et al., 2017). In particular, Kong et al. (2014) showed that Phil blocks activation of MAPKs and NF-κB in TNF-α–induced 3T3-L1 adipocytes. MAPKs and NF-κB pathways are well known to be essential for osteoclast formation and function. Given the wide clinical applications of Phil and its possible role in osteoclastogenesis, we hypothesized that Phil might represent a promising novel treatment for osteoclast-related diseases. Thus, we examined the effect of Phil on osteoclast formation and function *in vitro* and its capacity to protect against lipopolysaccharide (LPS)–induced osteolysis *in vivo* and subsequently investigated the underlying molecular mechanisms.

## Materials and Methods

### Reagents

Phil (C_27_H_34_O_11_, Purity ≥ 98%) was obtained from Chengdu Mansite Pharmaceutical Co. (Chengdu, Sichuan, China) (**Figure 1F**). Recombinant mouse M-CSF and RANKL were purchased from R&D Systems (Minneapolis, MN, USA). Dexamethasone, β-glycerophosphate, ascorbic acid, MTS reagents, tartrate-resistant acid phosphatase (TRAP) staining kit, and LPS were all gained from Sigma-Aldrich (St. Louis, MO, USA). Alkaline phosphatase (ALP) staining kit was obtained from Beyotime Biotechnology Inc. (Shanghai, China). TRIzol reagent was purchased from Invitrogen (Invitrogen Life Technologies, Carlsbad, CA, USA). Primary antibodies targeting phosphorylated extracellular signal-regulated kinase (p-ERK), phosphorylated c-Jun N-terminal kinases (p-JNK), phosphorylated p38 (p-p38), total ERK, total JNK, total p38, inhibitor of NF-κB (IκBα), and glyceraldehyde-3-phosphate dehydrogenase (GAPDH) were from CST (Cell Signaling Technology, Inc., Beverly, MA, USA). Antibodies including c-Fos and NFATc1 were purchased from BD Biosciences (San Jose, CA, USA).

### MTS Assay

The cell viability of bone marrow–derived macrophages (BMMs) was measured by an MTS assay. BMM cells (8×10^3^cells/well) were seeded into 96-well plates and then treated with complete MEM containing M-CSF (30 ng/ml) and various concentrations of Phil for 48h. After adding MTS/PMS mixture, the cells were continued to incubate for another 4h. The absorbance change at 490 nm was measured using a microplate reader (BMG LABTECH GmbH, Ortenberg, Germany). All data were obtained from at least three repeated experiments, and the results were expressed as a percentage of vehicle-treated control cells.

### *In Vitro* Osteoclastogenesis Assay

BMM cells were isolated, purified, and cultured as our previous reports (Liu et al., 2015; Li et al., 2018). These cells were plated into 96-well plates at a density of 8×10^3^ per well (in triplicate) and treated with various doses of Phil (0, 2.5, 5, 10, and 20µM) in the presence of M-CSF (30 ng/ml) and RANKL (100 ng/ml) for 5 days. The doses of Phil were determined based on the previous studies (Kong et al., 2014; Pan et al., 2014; Teng et al., 2014), MTS data, and our preliminary experiments. The mature osteoclasts were fixed with 4% paraformaldehyde and then stained for TRAP activity. The numbers and areas of TRAP^+^ multinucleated cell (nuclei ≥ 3) were determined using ImageJ software. The experiments were independently repeated three times.

### Apoptosis Assay

The effect of Phil on apoptosis was determined using an annexin V–fluorescein isothiocyanate/propidium iodide (PI) apoptosis detection kit (KenGEN Biotech. Co., Ltd., Nanjing, Jiangsu, China). BMM cells (1×10^6^ cells/well) were seeded into 6-well plates and then treated with complete MEM medium containing M-CSF (30ng/ml) and various doses of Phil (0, 5, 10, and 20µM) for 24h. The cells were washed with phosphate-buffered saline (PBS) and pelleted by centrifugation. After resuspending in binding buffer, the apoptotic cells were stained by annexin V and PI and detected using fluorescence-activated cell sorting (FACScan; Becton Dickinson, Franklin Lakes, NJ, USA).

### *In Vitro* Osteoblast Differentiation

Human osteoblasts (hFOB 1.19) were obtained from the Cell Bank of the Chinese Academy of Sciences (Shanghai, China). The hFOB 1.19 cells were cultured and induced to differentiation as our previous description (Liu et al., 2015). In brief, these cells were cultured in DMEM/F12 (Thermo Fisher Scientific, Inc.) supplemented with 10% fetal bovine serum (Thermo Fisher Scientific, Inc.), 100 U/ml penicillin-streptomycin (Sigma-Aldrich), and 0.3 mg/ml G418 (Sigma-Aldrich) in a humidified atmosphere of 5% CO_2_. For *in vitro* osteoblastogenesis, hFOB 1.19 cells were incubated in osteogenic inducing medium containing 10nM dexamethasone, 10mM β-glycerophosphate, and 50µg/ml ascorbic acid in the presence of various concentrations of Phil for 10days. The cells were fixed with 4% paraformaldehyde and then stained for ALP activity.

### Resorption Pit Assay

Fresh bovine femur was obtained from a local butcher. After removing the muscles and washing several times with PBS, the cortical bone was cut into 100-µm-thick slices using a Buhler Isomet low-speed saw (Buehler Ltd., Lake Bluff, IL, USA). The bone slices were sonicated, washed with MilliQ water, and then sterilized with 70% ethanol for 2 minutes. After rinsing with sterile PBS and incubating with α-MEM for 4 h, the bone slices were put into the 96-well plates and used for subsequent experiments. BMMs (8×10^3^ cells/well) were plated on theses bone slices and stimulated with M-CSF (30ng/ml) and RANKL (100ng/ml) for 3 to 4 days. When mature osteoclasts began to form, different doses of Phil were added to the wells and continuously cultured for 2 days. TRAP staining was performed, and the TRAP^+^ multinucleated cells (nuclei ≥ 3) were quantified. After removal of the cells, the bone slices were visualized by scanning electron microscopy (JSM 5610 LV; JEOL, Tokyo, Japan). Five view fields per bone slice were randomly selected, and bone resorption area was calculated using Image Pro-Plus 5.0 software (Media Cybernetics, Silver Spring, MD, USA). The experiments were repeated at least three times.

### Actin Ring-Formation Assay

F-actin ring-formation assay was performed according to our previous report (Li et al., 2018). In brief, after pretreating with varying doses of Phil for 2 days, the mature osteoclasts cultured on bone slices were fixed with 4% paraformaldehyde for 15 min and then permeabilized with 0.5% Triton X-100 for 10 min. F-actin rings were stained with rhodamine-conjugated phalloidin for 15 min and the nuclei with 4′, 6-diamidino-2-phenylindole (DAPI) dye. The fluorescence images were taken with a Nikon A1R resonance scanning confocal microscope (Nikon, Tokyo, Japan). The number and size of F-actin rings were analyzed using ImageJ software (National Institutes of Health, Bethesda, MD, USA).

### Quantitative Polymerase Chain Reaction Analysis

We used real-time polymerase chain reaction (PCR) to detect the expression of osteoclast-specific genes in mature osteoclasts, RANKL expression in osteoblasts, and cytokines in calvarias. For osteoclast-specific gene expression, BMMs (4×10^4^ cells/well) were seeded in 12-well plates and cotreated with M-CSF, RANKL, and different concentrations of Phil (0, 5, 10, and 20µM) for 5 days. For RANKL expression in osteoblasts, hFOB 1.19 cells were treated with LPS (100ng/ml) and different doses of Phil (0, 5, 10, and 20µM) for 24h. For cytokine analysis, calvarias from *in vivo* experiments were homogenized, and cytokines including TNFα and RANKL were measured. After the samples were prepared, total RNA was extracted using TRIzol reagent, and cDNA was subsequently synthesized from 500ng of total RNA using reverse transcriptase (TaKaRa Biotechnology Co., Ltd., Dalian, Shenyang, China). Real-time PCR was performed using a SYBR Premix Ex Tag kit (TaKaRa Biotechnology) and Mastercycler ep realplex 2 systems (Eppendorf, Hamburg, Germany). The following primer sets were used: mouse *cathepsin K* (*CTSK*), 5′-CTTCCAATACGTGCAGCAGA-3′ (forward), 5′-TCTTCAGGGCTTTCTCGTTC-3′ (reverse); mouse *TRAP*, 5′-CTGGAGTGCACGATGC CAGCGACA-3′ (forward), 5′-TCCGTGCTCGGCGATGGACCAGA-3′ (reverse); mouse *calcitonin receptor* (*CTR*), 5′-TCAGGAACCACGGAATCCTC-3′ (forward), 5′-ACATTCAAGCGGATGCGTCT-3′ (reverse); mouse *ATPase H^+^* *transporting V0 subunit d2* (*ATP6V0d2*), 5′-GTGAGACCTTGGAAGACCTGAA‐3′ (forward), 5′-GAGAAATGTGCTCAGGGGCT‐3′ (reverse); mouse *matrix metallopeptidase 9* (*MMP9*), 5′-CGTGTCTGGAGATTCGACTTGA‐3′ (forward). 5′-TTGGAAACTCACACGCCAGA-3′ (reverse); mouse *c-Fos*, 5′-CCAGTCAAGAGCATCAGCAA-3′ (forward), 5′-AAGTAGTGCAGCCCGGAGTA-3′ (reverse); mouse *NFATc1*, 5′-CCGTTGCTTCCAGAAAATAACA-3′ (forward), 5′-TGTGGGATGTGAACTCGGAA-3′ (reverse); mouse *RANKL*, 5′-CTGATGAAAGGAGGGAG-3′ (forward), 5′-ATCCAGCAGGGAAGGGT-3′ (reverse); mouse *TNFα*, 5′-GTGAAGGGAATGGGTGTT-3′ (forward), 5′-GGTCACTGTCCCAGCATC-3′ (reverse); mouse *GAPDH*, 5′-ACCACAGTCCAAGCCATCAC-3′ (forward), 5′- CACATTGGGGGTAGGAACAC-3′ (reverse); human *RANKL*, 5′- TCGTTGGATCACAGCACATCA-3′ (forward), 5′-TATGGGAACCAGATGGGATGTC-3′ (reverse); human β*-actin*, 5′-AGCGAGCATCCCCCAAAGTT-3′ (forward), 5′-GGGCACGAAGGCTCATCATT-3′ (reverse). Three independent experiments were conducted.

### Western Blot Analysis

To determine the effect of Phil on MAPK and NF-κB signaling pathways, BMMs (5×10^5^cells/well) were plated in 12-well plates and pretreated with various concentrations of Phil (0, 5, 10, and 20µM) for 4h. RANKL was then added to stimulate for 15 min. To detect Phil’s effect on the protein expression of c-Fos and NFATc1, BMMs were treated with 10µM Phil in the presence of RANKL (100ng/ml) and M-CSF (30ng/ml) for 0, 1, 3, or 5days. The cells were lysed with radioimmunoprecipitation assay buffer, and the protein was collected by centrifugation. After separating by sodium dodecyl sulfate–polyacrylamide gel electrophoresis, the protein was transferred to polyvinylidene difluoride membranes and incubated with specific antibodies including p-p38 (dilution 1:1,000), p-JNK (dilution 1:1,000), p-ERK (dilution 1:1,000), p38 (dilution 1:1,000), JNK (dilution 1:1,000), ERK (dilution 1:1,000), IκB (dilution 1:1,000), c-Fos (dilution 1:1,000), NFATc1 (dilution 1:1,000), and GAPDH (dilution 1:1,000). The protein bands were detected using enhanced chemiluminescence reagents (Amersham, Shanghai, China), and the relative expression of each protein was analyzed using the ImageJ software.

### LPS-Induced Calvarial Osteolysis Model

The animal experiments were approval by the Experimental Committee of Nanjing Normal University (#2019057, approved date June 18, 2019). Twenty-eight healthy male C57BL/6 mice (8weeks old) were purchased from Shanghai SLAC Laboratory Animal Co., Ltd. (Shanghai, China). Mice were housed under specific pathogen-free conditions (22°C, 50%–55% humidity, 12/12-h light/dark) with free access to water and food.

LPS-induced calvarial osteolysis model was established as the previous studies (Fu et al., 2019; Wu et al., 2019). In brief, the animals were randomly divided into four groups (n = 10): PBS control (sham), LPS injection (5 mg/kg body weight) (vehicle), and LPS together with different concentrations of Phil (5 and 10mg/kg). The concentrations of Phil were determined based on the previous studies (Wei-Ting et al., 2013; Qu et al., 2016; Cheng et al., 2017) and our preliminary experiments. The sham group was subcutaneously injected with 100µL PBS, and the other groups were injected with 100µl LPS over the sagittal midline suture of the calvaria. Phil or vehicle was intraperitoneally injected every other day for a 7-day period. At the end of the experiments, the mice were sacrificed, and the calvarias were dissected for further assessment including micro–computed tomography (micro-CT) scanning, histological analysis, and cytokine detection.

### Micro-CT Scanning

To assess the degree of osteolysis, the calvarias were scanned using a high-resolution micro-CT (SkyScan1176; Bruker, Germany) with an isometric resolution of 9µm. After reconstruction of the whole calvaria, the round region of interest around the midline suture (4-mm diameter) was chosen for further quantitative analysis. Bone mineral density (BMD) and bone volume/tissue volume (BV/TV) were measured using the CTan program (SkyScan; Kontich, Belgium).

### Histological and Histomorphometric Analysis

After micro-CT scanning, the calvarias were decalcified with 10% EDTA, embedded in paraffin, and stained with hematoxylin and eosin (H&E) and TRAP. The histomorphometric parameters including the percentage of infiltrated fibrotic area against total tissue area (erosion area, %), the number of TRAP^+^ osteoclasts normalized to bone area, and the percentage of osteoclast surface per bone surface (OcS/BS, %) were calculated with Image Pro-Plus 5.0 software (Media Cybernetics, Silver Spring, MD, USA).

### Statistical Analysis

Results were expressed as the mean ± SD with three or more independent experiments. Statistical difference was determined using one-way analysis of variance, followed by Tukey *post hoc* analysis. *P*<0.05 was designated as statistically significant.

## Results

### Phil Significantly Inhibited RANKL-Induced Osteoclast Formation

**Figure 2:**
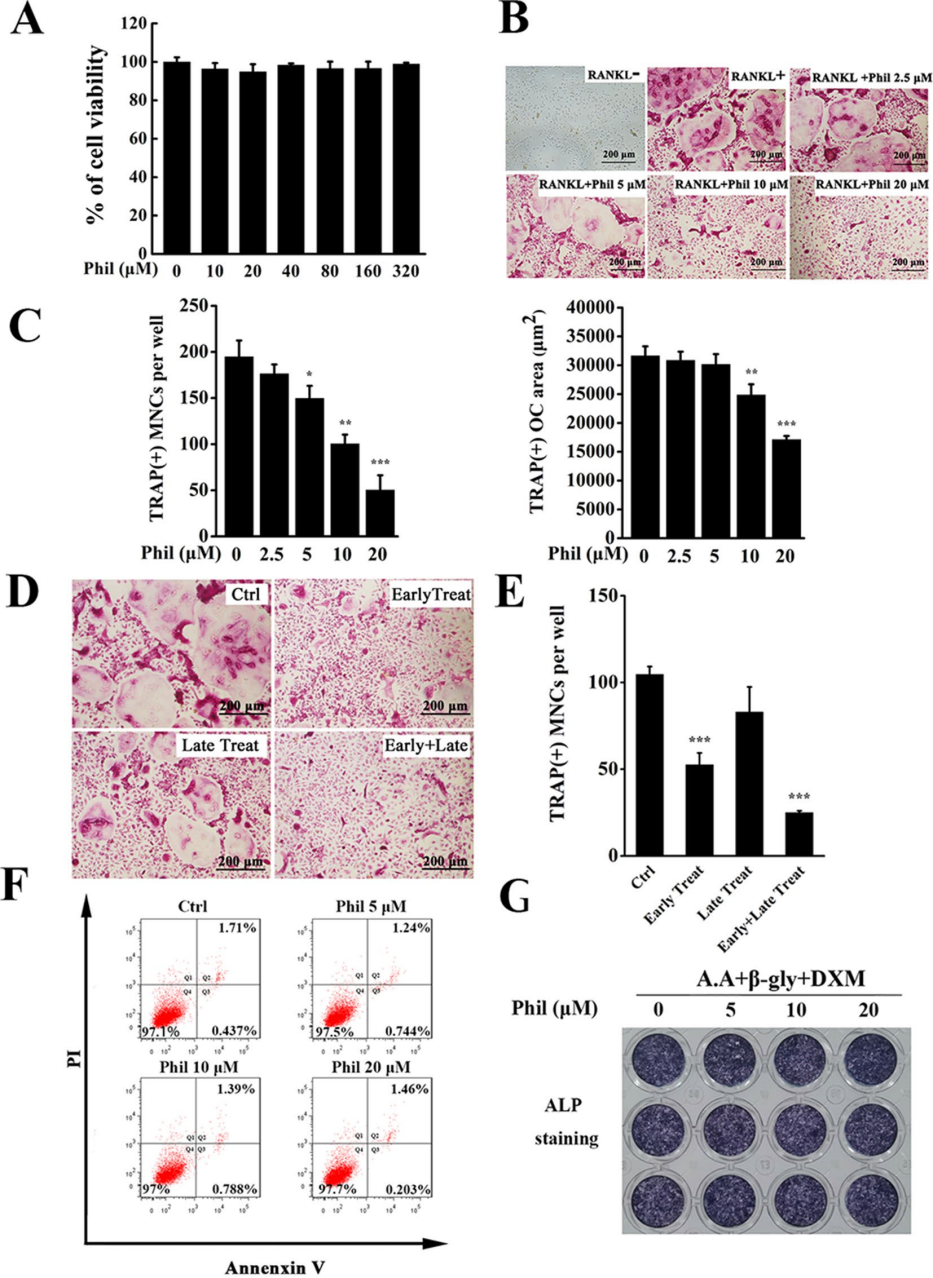
Phil significantly and dose-dependently inhibits RANKL-induced osteoclast differentiation. **(A)** MTS assay was performed to measure the viability of BMM cells. **(B)** BMM cells were seeded into 96-well plates and treated with various doses of Phil (0, 2.5, 5, 10, and 20µM), followed by stimulation with M-CSF (30ng/ml) and RANKL (100ng/ml) for 5 days. After fixing with 4% paraformaldehyde, the cells were stained for TRAP activity. **(C)** The number and area of TRAP^+^ cells (≥3 nuclei) were counted. n = 3, *P<0.05, **P<0.01 and ***P<0.001 relative to RANKL-induced, Phil-untreated control. **(D)** Addition of Phil (10µM) at different differentiation time points (Ctrl: RANKL stimulation for 5 days without Phil; Early Treat: coincubation with RANKL and Phil for 2 days and RANKL continuous incubation for another 3 days; Late Treat: RANKL incubation for 2 days and subsequent coincubation with RANKL and Phil for 3 days; Early+Late Treat: coincubation with RANKL and Phil for 5 days). TRAP staining was carried out to quantify the number of TRAP^+^ osteoclasts (≥3 nuclei). The data were shown in **(E)**. n = 3, ***P<0.001 relative to control. **(F)** After treating the cells with varying doses of Phil (0, 5, 10, and 20µM) for 24 h, flow cytometry was used to detect apoptosis. **(G)** Human osteoblasts (hFOB 1.19) were treated with different concentrations of Phil in the presence of ascorbic acid (A.A), β-glycerophosphate (β-gly), and dexamethasone (DXM) for 10 days. The cells were fixed with 4% paraformaldehyde and then stained for alkaline phosphatase (ALP) activity.

An *in vitro* MTS-based screen of compounds allowed us to identify Phil as an agent that inhibited BMM proliferation and survival. As shown in **Figure 2A**, no cytotoxicity was detected, even at dose as high as 320µM. Since BMMs are osteoclast progenitors, we next explored the effect of Phil on RANKL-induced osteoclastogenesis. TRAP staining results showed that numerous TRAP^+^ osteoclasts were observed in the control group. However, the number and area of osteoclast were significantly suppressed by Phil treatment in a dose-dependent manner (**Figures 2B, C**). To determine at which stages Phil suppressed osteoclast formation, 10µM Phil was added at different differentiation time points, as our previous report (Li et al., 2018). As shown in **Figures 2D, E**, Phil treatment on day 3 of the 5-day culture (late treatment) had little effect on osteoclast formation. However, Phil treatment on the first 2days of RANKL induction (early treatment) or Phil treatment at two stages (early and late treatment) significantly reduced the number of osteoclasts (**Figures 2D, E**). These data suggested that, to ensure the inhibitory effect on osteoclastogenesis, Phil should exist during the early stages or throughout the whole differentiation process. To exclude the possibility that the inhibition of Phil on osteoclastogenesis was due to apoptosis, flow cytometry was used to analyze the apoptosis-inducing action of Phil. The results showed that the given doses of Phil did not affect apoptosis (**Figure 2F**).

Since the maintenance of bone homeostasis is dependent on the balance between bone-resorbing osteoclasts and bone-forming osteoblasts, we next assessed the effect of Phil on osteoblast formation using *in vitro* osteoblastogenesis assay. As shown in **Figure 2G**, the expression of ALP was not significantly different between control and Phil-treated groups, indicating that Phil at the given doses did not affect osteoblast differentiation.

### Phil Significantly Impaired Osteoclastic Resorptive Activity and F-Actin Ring Formation

**Figure 3:**
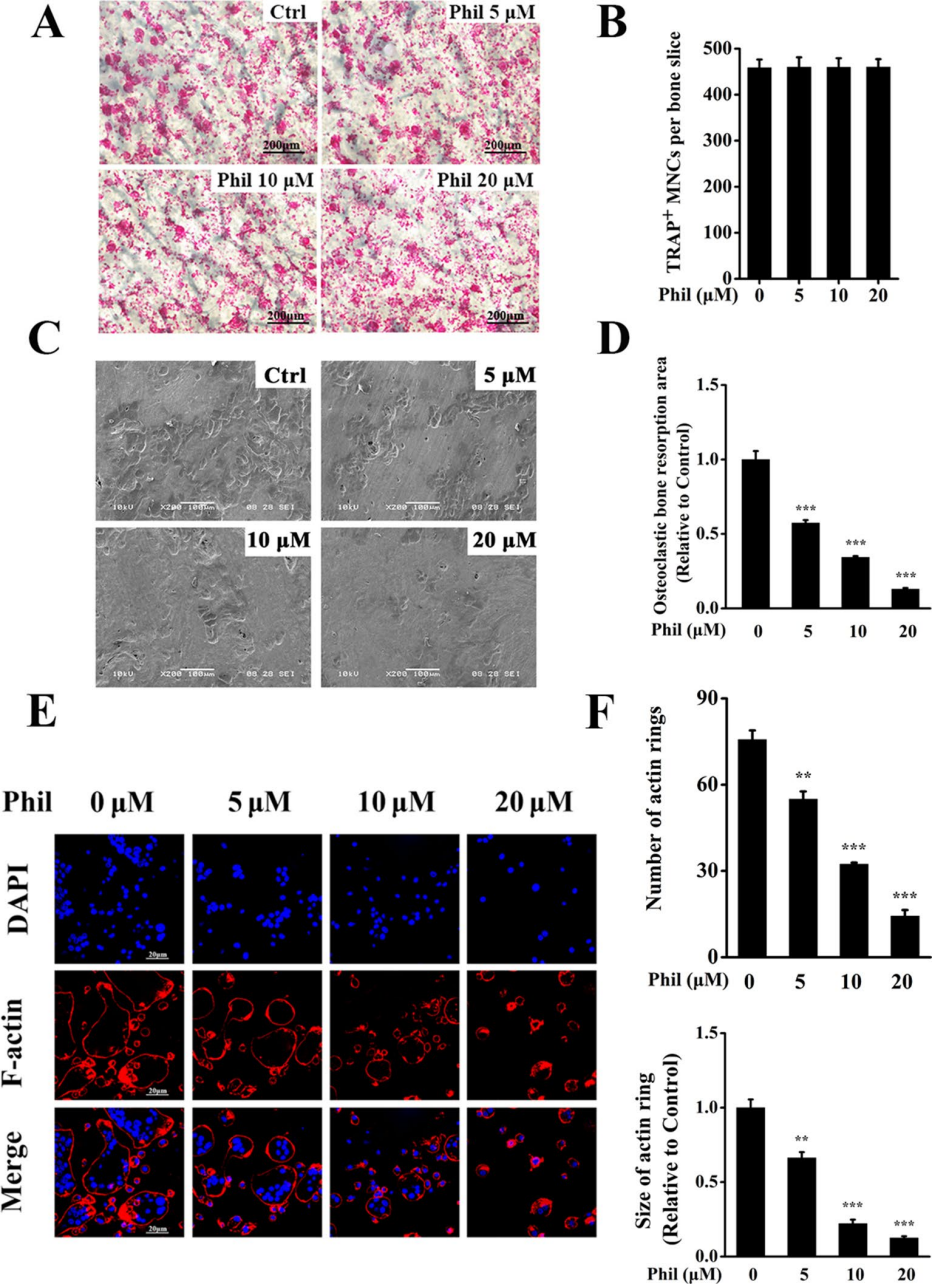
Phil impairs osteoclastic resorptive activity and F-actin ring formation. **(A)** Mature osteoclasts cultured on the bone slices were treated with different concentrations of Phil and then stained for TRAP activity. **(B)** The quantitative analysis of the number of TRAP^+^ osteoclasts (≥3 nuclei) from **(A)** indicated that Phil had no cytotoxic effect on mature osteoclasts. **(C)** After removal of the cells on bone slices, the resorption pits were visualized by scanning electron microscopy. **(D)** The area of resorption pit was calculated using ImageJ software (n = 3). ***P<0.001 relative to RANKL-induced, Phil-untreated group. **(E)** The effect of Phil on F-actin ring formation was assessed using immunofluorescent staining. **(F)** The number and size of F-actin rings were calculated (n = 3). **P<0.01 and ***P<0.001 relative to RANKL-induced, Phil-untreated group.

Since Phil inhibited osteoclast differentiation, we next investigated the effect of Phil on osteoclastic bone resorptive function. Mature osteoclasts cultured on the bone slices were treated with varying doses of Phil and subsequently stained for TRAP activity. As shown in **Figures 3A, B**, Phil did not affect the number of TRAP^+^ cells, indicating that Phil had no cytotoxic effect on mature osteoclasts. Scanning electron microscopy results showed that a large amount of bone resorption pits were formed on the control bone slices. However, Phil significantly and dose-dependently reduced the area of bone resorption, suggesting that Phil suppressed osteoclastic bone resorptive function *in vitro* (**Figures 3C, D**).

F-actin ring is indispensable for osteoclastic bone resorption. To further confirm the inhibition of Phil on bone resorption, F-actin rings were examined by immunofluorescence staining. As shown in **Figures 3E, F**, intact-structured F-actin rings were observed in the control group. However, Phil treatment significantly decreased the number and size of F-actin rings in a dose-dependent manner. These results were consistent with the data obtained from bone resorption pit assay.

### Phil Significantly Suppressed the Expressions of RANKL-Induced Osteoclastic Marker Genes

**Figure 4:**
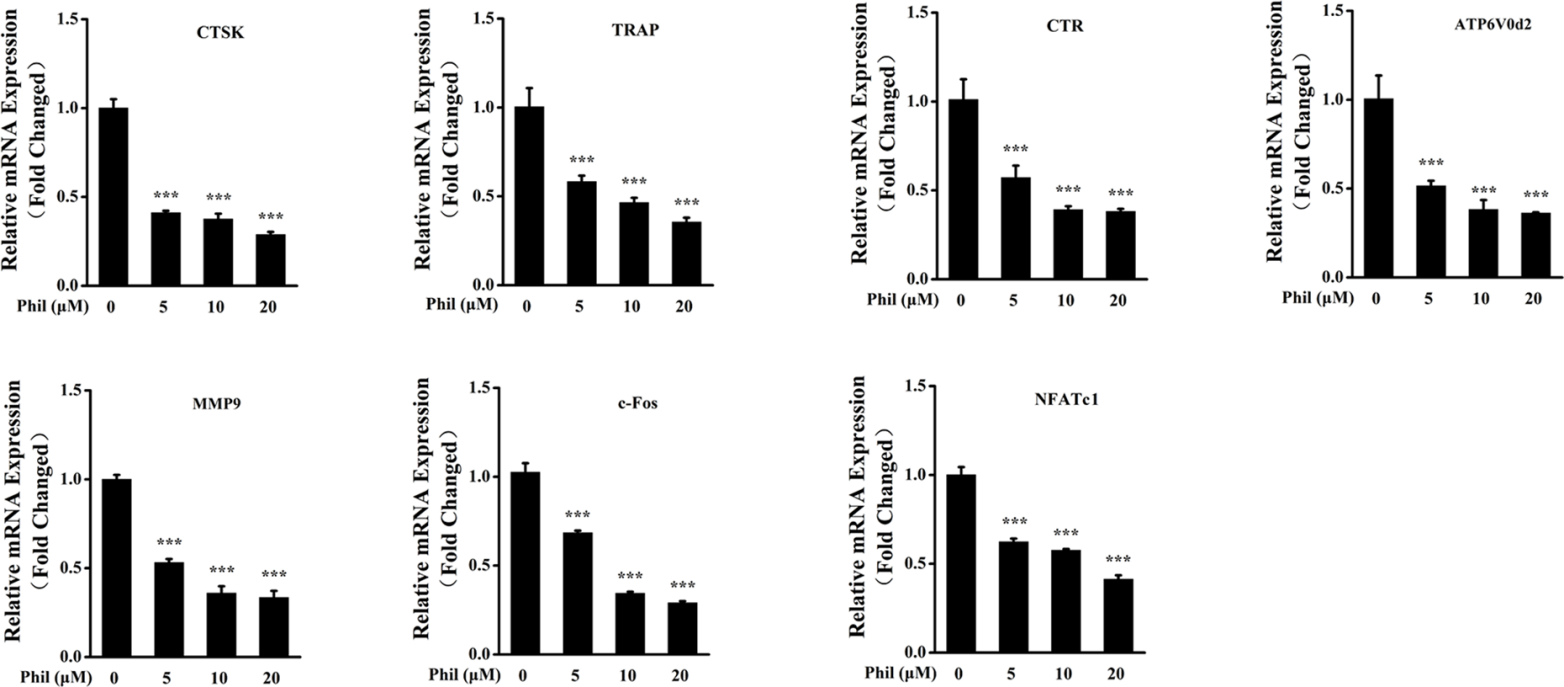
Phil decreases the transcript levels of RANKL-stimulated osteoclastic marker genes. BMM cells were cotreated with different doses of Phil in the presence of RANKL (100ng/ml) and M-CSF (30ng/ml) for 5 days. Real-time PCR was then performed to examine the transcripts of osteoclastic marker genes. The mRNA levels of these genes were normalized to *GAPDH* (n = 3). ***P<0.001 relative to RANKL-induced, Phil-untreated group. CTSK, cathepsin K; TRAP, tartrate-resistant acid phosphatase; CTR, calcitonin receptor; ATP6V0d2, ATPase H^+^ transporting V0 subunit d2; MMP9, matrix metallopeptidase 9; NFATc1, nuclear factor of activated T-cells cytoplasmic 1 GAPDH, glyceraldehyde-3-phosphate dehydrogenase.

To further investigate the inhibitory effect of Phil on osteoclast formation and function, the transcript levels of osteoclastic marker genes including *CTSK*, *TRAP*, *CTR*, *ATP6V0d2*, *MMP9*, *c-Fos*, and *NFATc1* were examined. As shown in **Figure 4**, the expressions of these osteoclast-specific genes were up-regulated under RANKL stimulation. However, these up-regulations were significantly suppressed by Phil treatment, which further confirmed the inhibitory action of Phil on osteoclast formation and function.

### Phil Significantly Blocked RANKL-Induced JNK and ERK Activation

**Figure 5:**
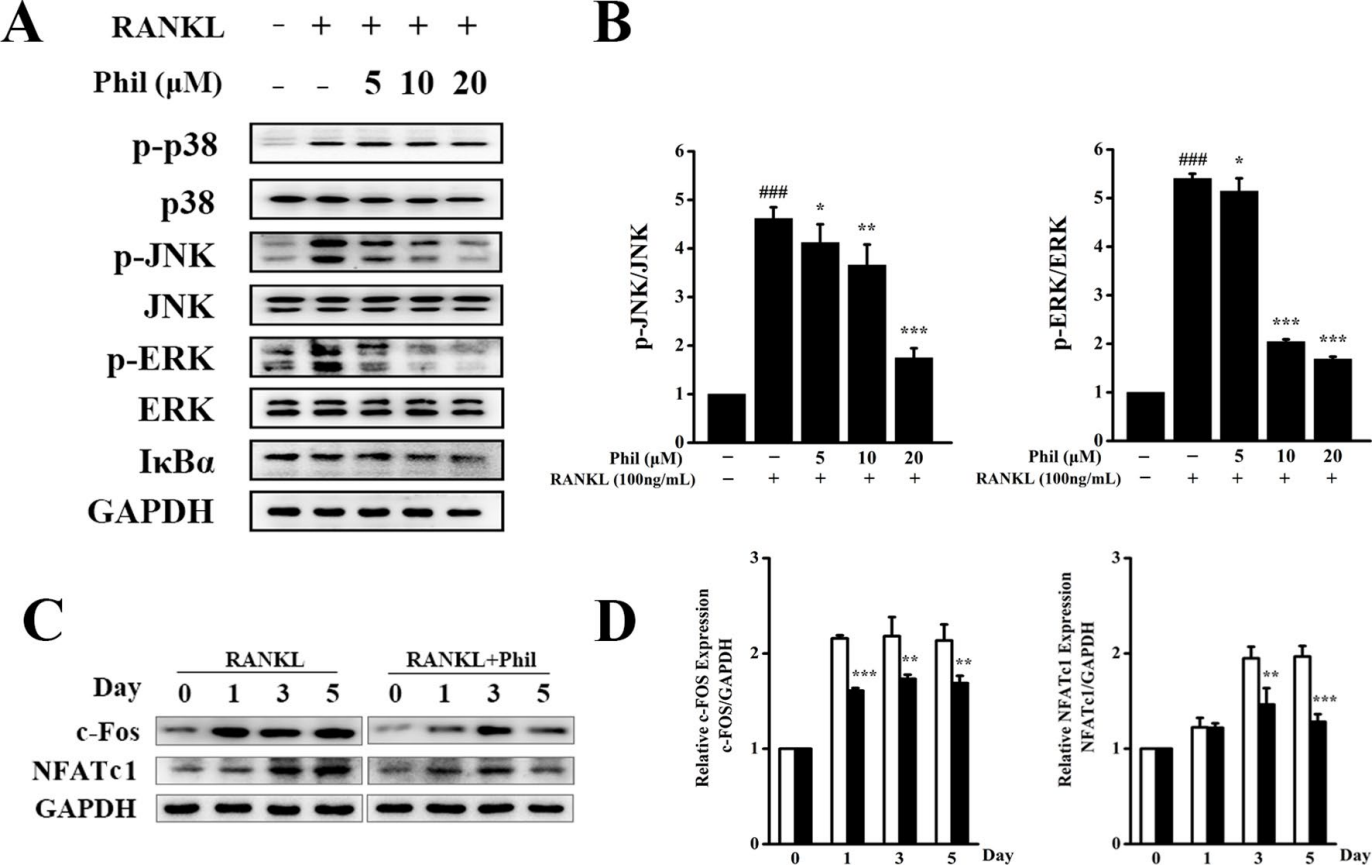
Phil inhibits RANKL-stimulated JNK and ERK activations. **(A)** After treating BMM cells with different doses of Phil for 4 h, RANKL (100ng/ml) was added to stimulate for 15 min. Protein was then extracted for Western blot with the indicated antibodies. **(B)** The ratios of p-JNK/JNK and p-ERK/ERK were determined using ImageJ software. n = 3, ^###^*P*<0.001 relative to RANKL-untreated, Phil-untreated group; *P<0.05, **P<0.01 and ***P<0.001 relative to RANKL-induced, Phil-untreated group. **(C)** BMM cells were treated with Phil (10µM) for 0, 1, 3, or 5 days. Western blot was used to examine the protein levels of c-Fos, NFATc1, and GAPDH. **(D)** The ratios of c-Fos/GAPDH and NFATc1/GAPDH were analyzed using ImageJ software. n = 3, **P<0.01 and ***P<0.001 relative to RANKL-induced, Phil -untreated group.

To unveil the molecular mechanisms through which Phil exerted an inhibitory effect on osteoclastogenesis, RANKL-induced MAPKs and NF-κB signaling pathways were investigated. As shown in **Figures 5A, B**, after 15 min of RANKL stimulation, the phosphorylations of JNK and ERK were significantly increased. These increases were significantly inhibited by Phil in a dose-dependent manner. However, Phil had little impact on the phosphorylation of p38. Also, Phil treatment did not affect the degradation of IκBα, suggesting that the inhibition of Phil on osteoclastogenesis was not associated with NF-κB signaling pathway.

It has been reported that activation of the MAPKs pathways can induce c-Fos and NFATc1 expression, which are pivotal downstream transcription factors during osteoclast differentiation and function. Thus, we used Western blot to assess the protein expression of c-Fos and NFATc1. As shown in **Figures 5C, D**, RANKL stimulation increased the protein levels of c-Fos and NFATc1. However, these increases were effectively suppressed by Phil treatment, which is consistent with the data from real-time PCR.

### Phil Protected Against LPS-Induced Osteolysis

**Figure 6:**
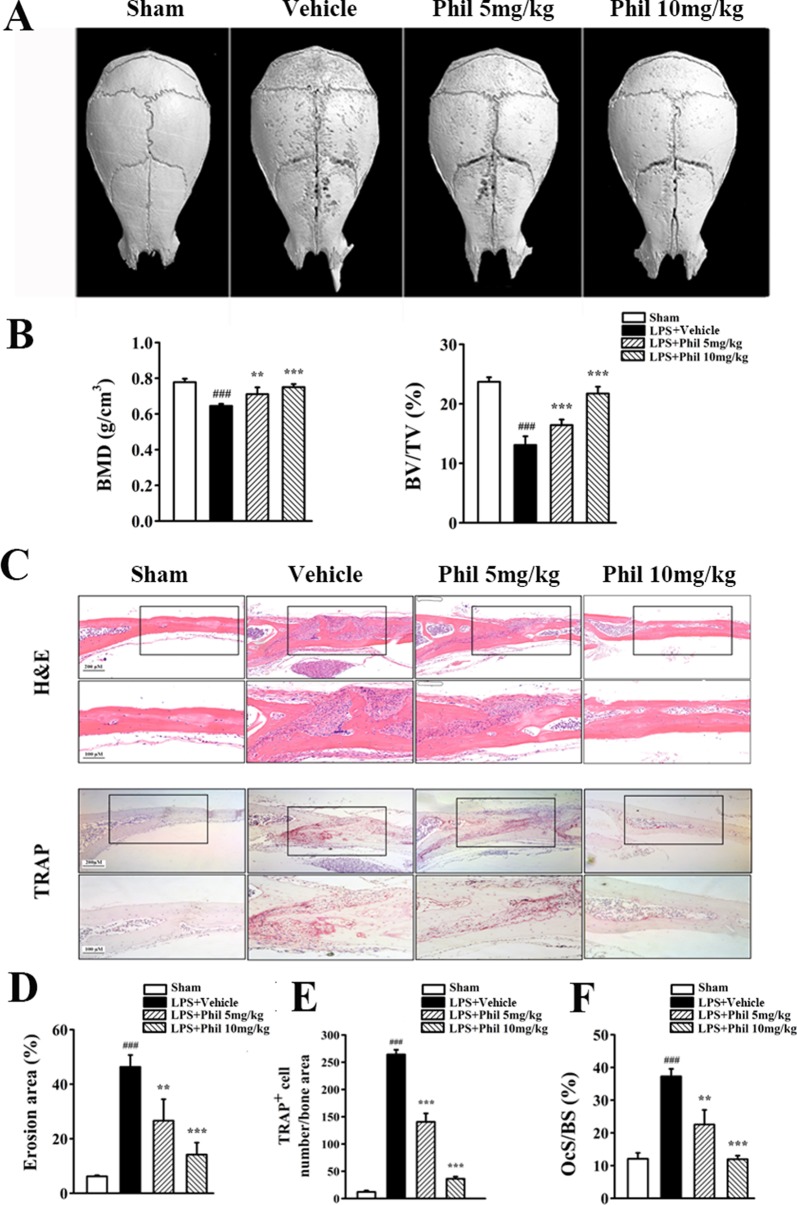
Phil inhibits LPS-induced osteolysis in mice. **(A)** Representative 3D reconstruction of the whole calvarias from different groups. **(B)** The quantitative analysis of bone mineral density (BMD) and bone volume/tissue volume (BV/TV) (n = 7). ^###^*P*<0.001 relative to the sham group; **P<0.01 and ***P<0.001 relative to the vehicle group. **(C)** Representative images of calvarial sections stained with H&E and TRAP from different groups. **(D–F)** Histomorphometric analysis of the percentage of infiltrated fibrotic area against total tissue area (erosion area, %), the number of TRAP^+^ osteoclasts normalized to bone area, and the percentage of osteoclast surface per bone surface (OcS/BS, %) (n = 7).

Our *in vitro* studies demonstrated that Phil possessed antiosteoclastogenic and antiresorptive capabilities. We next used an LPS-induced calvarial osteolysis model to further explore the potential protective effects of Phil *in vivo*. As shown in the three-dimensional reconstruction images in **Figure 6A**, compared to the sham group, extensive erosion was observed on the bone surface of the vehicle group. However, the severity of LPS-induced osteolysis was significantly inhibited by Phil treatment (**Figure 6A**). The quantitative analysis of bone parameters showed that LPS induction resulted in significantly decreased BMD and BV/TV (**Figure 6B**). However, these decreases were significantly and dose-dependently inhibited by Phil treatment (**Figure 6B**). In line with the micro-CT data, H&E staining and histomorphometric assessment showed that Phil significantly reduced LPS-induced bone erosive areas, which further confirmed the protection of Phil against inflammatory bone destruction (**Figures 6C, D**). As expected, the number of TRAP^+^ osteoclasts and OcS/BS were significantly decreased by Phil treatment in a dose-dependent manner (**Figures 6C, E, F**), suggesting that the osteolysis-inhibiting effect of Phil was due to, at least in part, its suppression on osteoclastogenesis.

### Phil Suppressed LPS-Induced Osteoclast-Related Cytokine Expression *In Vivo* and in Cultured Osteoblasts

**Figure 7:**
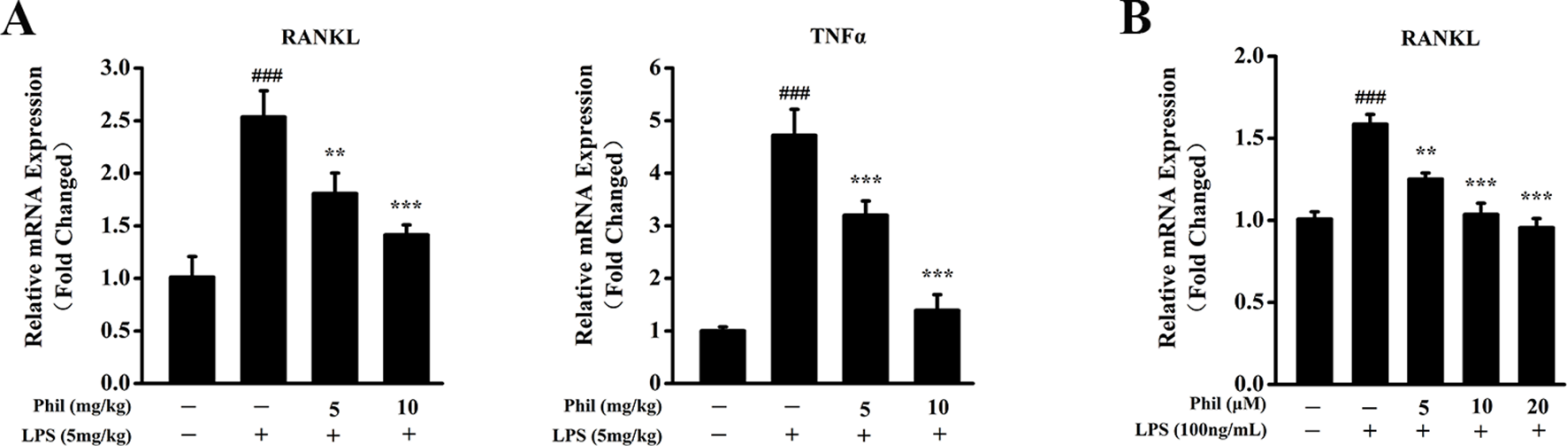
Phil decreases LPS-induced osteoclast-related cytokine expression in calvarias and in cultured osteoblasts. **(A)** The calvarias from *in vivo* experiments were homogenized and the expressions of *RANKL* and *TNF*α were determined by real-time PCR. The mRNA levels of these two genes were normalized to *GAPDH* (n = 3). ^###^*P*<0.001 relative to the sham group; **P<0.01 and ***P<0.001 relative to the vehicle group. **(B)** The hFOB 1.19 cells were treated with LPS (100ng/ml) and different doses of Phil (0, 5, 10, and 20µM) for 24h. The mRNA level of *RANKL* was determined by real-time PCR and normalized to *β-actin* (n = 3). ^###^*P*<0.001 relative to RANKL-untreated, Phil-untreated group; **P<0.01 and ***P<0.001 relative to RANKL-induced, Phil-untreated group.

To investigate whether the suppressing effect of Phil on LPS-induced osteoclast formation *in vivo* was due to its inhibition on osteoclast-related cytokine expression, real-time PCR was performed to examine the mRNA levels of *RANKL* and *TNFα* in calvarias. As shown in **Figure 7A**, *RANKL* and *TNFα* expressions were significantly increased in the LPS group compared with the sham group. However, these increases were significantly suppressed by Phil treatment (**Figure 7A**). It is well known that RANKL is the most important cytokine for osteoclastogenesis, and LPS can stimulate osteoblast to produce and expression osteoclast-related cytokine RANKL (Kikuchi et al., 2001; Wei and Zvi, 2010). Thus, we examined the mRNA expression of *RANKL* in osteoblasts *in vitro*. As shown in **Figure 7B**, LPS induction effectively up-regulated *RANKL* expression in hFOB 1.19 cells. However, Phil treatment significantly suppressed this increased expression (**Figure 7B**).

## Discussion

Overactivated osteoclastogenesis is the main reason of a lot of lytic bone disorders (Tolar et al., 2004; Zannettino et al., 2005; Liu et al., 2017). Thus, suppressing osteoclast formation and function is considered as one of the most important strategies for treating the osteolytic diseases. Although some osteoclast-target drugs such as bisphosphonates have certain curative effects, their toxic and side effects can cause adverse reactions or inaccurate curative effects (Eriksen et al., 1985; Hasling et al., 1987). Thus, searching for alternative and well-tolerated antiosteoclastogenesis drugs is still urgently required.

Forsythia plants are widely used in daily life and clinical medicines because of their diverse biological activities, definite curative effects, abundant resources, and less adverse effects. Here in this study, we showed that Phil, an active ingredient extracted from *F. suspensa*, could effectively attenuate LPS-induced bone boss *in vivo* by directly inhibiting osteoclast formation and inhibiting LPS-induced RANKL production in osteoblasts.

Lipopolysaccharide, a membrane component of gram‐negative bacteria, has long been used as a potent inducer of osteolytic bone loss (Kong et al., 2017). Studies suggested that bacterial endotoxins that contaminate prostheses are primary contributors to aseptic periprosthetic osteolysis by enhancing the reactivity of wear particles and inflammatory response (Zaveri et al., 2017). Among them, LPS is the best known. It is capable of stimulating the immune cells and osteoblasts to produce proinflammatory and pro-osteoclastogenic cytokines. These cytokines can promote osteoclast formation and activation, which further lead to osteolysis or bone loss (Bi et al., 2010; Hitoshi et al., 2010). Our *in vivo* studies showed that the administration of Phil significantly and dose-dependently suppressed LPS-induced bone loss, as illustrated by enhanced BMD and BV/TV and decreased erosion area. Phil treatment was accompanied by a reduced number of TRAP^+^ osteoclasts as well as OcS/BS. With respect to this inhibitory effect of Phil on osteoclast formation *in vivo*, the following two possible mechanisms should be taken into consideration. One possible mechanism is that Phil suppresses LPS-induced osteoclast-related cytokine expression. It has been reported that LPS can induce RANKL and TNFα expression *in vivo* (Nair et al., 1996; Zou et al., 2003; Naohisa et al., 2004). And these two cytokines are well known to be crucial for osteoclast formation and function. Thus, we examined the expression of *RANKL* and *TNFα* in calvarias. As expected, Phil significantly inhibited *RANKL* and *TNFα* mRNA levels *in vivo*. Furthermore, our *in vitro* study further suggested that the down-regulation of *RANKL* might result from a direct inhibition of Phil on osteoblasts. Another possible mechanism of Phil’s inhibition is that Phil exerts direct effects on osteoclast formation and bone resorption. Indeed, our *in vitro* osteoclastogenesis assay showed that Phil significantly suppressed RANKL-induced osteoclast number and area, which was further confirmed by the data of osteoclastic marker gene expression. Also, resorption pit assay and F-actin ring-formation assay demonstrated that Phil significantly impaired osteoclastic resorptive activity. Taken together, all these results indicated that the inhibitory effects of Phil on osteoclast formation *in vivo* are due to both decreased production of osteoclast-associated cytokines and direct actions of Phil on osteoclast differentiation and function. Additionally, although we did not examine the effect of Phil on osteoblastic bone formation *in vivo*, our *in vitro* studies indicated that Phil at the given doses did not affect osteoblast differentiation.

After confirming that Phil plays inhibitory roles in LPS-induced osteolysis model and RANKL-induced osteoclastogenesis, we next explored the underlying molecular mechanism. RANKL-induced MAPKs pathways, namely, ERK, JNK, and p38, are well known to be essential for normal osteoclast differentiation and activity (Tanaka et al., 2003; Ritchlin et al., 2004). Phosphorylated ERK has been found to translocate into nucleus to further phosphorylate and activate AP-1 (Fos and Jun) and NFAT proteins, thereby inducing osteoclast formation (Soo Woong et al., 2002; Anne et al., 2010). BMM cells carrying a mutated form of c-Jun or lacking JNK1 show impaired osteoclast formation and resorptive activity (David et al., 2002; Ikeda et al., 2004). In addition, suppression of JNK activation at the prefusion osteoclast stage can lead to the reverse of TRAP-positive cells to TRAP-negative cells, which demonstrates that the JNK pathway is needed to maintain osteoclastic commitment and further promote osteoclastogenesis (Chang et al., 2008). The signaling cascade of p38 has been proven to be predominantly involved in osteoclast differentiation rather than the bone resorptive function of activated osteoclasts (Xiaotong et al., 2002; Lee et al., 2016). In our study, Western blot results showed that all the three MAPK members were dramatically induced by RANKL stimulation. Phil significantly and dose-dependently attenuated phosphorylation and therefore activation of JNK and ERK, but had little effect on p38 activation. In addition to MAPKs pathways, we also examined the effect of Phil on NF-κB pathway, which has been demonstrated to play a pivotal role in osteoclastogenesis and bone resorption (Yamashita et al., 2007; Abuamer et al., 2008; Soysa et al., 2009). The results showed that Phil had no obvious effect on IκB degradation, which was not in consistence with the data of Kong et al. (2014) which found that Phil blocked TNFα–induced NF-κB pathway through inhibiting IKK phosphorylation and IκBα degradation. This discrepancy may arise from the different stimuli and different cell types. Another explanation to the lack of effect of Phil on NF-κB pathway may be lying on the stimulation time of RANKL used in this study. Since our studies primarily focused on investigating the dose-dependent effect of Phil on IκBα degradation, we only examined the level of IκBα at 15 min of RANKL stimulation. We cannot exclude the possibility that Phil may affect IκBα degradation at other stimulation time points. Further studies are needed to gain a clearer understanding of the underlying molecular mechanism of Phil’s action.

NFATc1 is considered as an indispensable downstream transcription factor of RANKL-induced signaling pathways, including JNK and ERK (Asagiri et al., 2005; Takayanagi, 2010). Consistent with the attenuated activation of MAPKs (JNK and ERK), the expression of NFATc1 at mRNA and protein levels was significantly suppressed by Phil treatment. This suppression was further confirmed by the decreased expression of NFATc1’s upstream regulator c-Fos and its downstream osteoclastic marker genes, such as *CTSK*, *TRAP*, *CTR*, *ATP6V0d2*, and *MMP9*. Additionally, since the antiosteoclastogenic action of Phil occurred at the early stage of osteoclast formation, we speculated that the inhibition of Phil on NFATc1’s expression was mainly dependent on the indirect effect through suppressing the early signaling pathways (JNK and ERK), but not the direct effect.

In conclusion, our studies demonstrated for the first time that Phil effectively inhibited RANKL-induced osteoclast formation and bone resorption *in vitro* and protected against LPS-induced osteolysis *in vivo*. The inhibitory effects of Phil could be mediated by the early MAPK signaling cascades (JNK and ERK), which down-regulated the expression of NFATc1 and NFATc1-mediated downstream osteoclastic marker genes. These findings suggested that Phil might be a promising candidate for the treatment of osteoclast-related osteolytic diseases.

## Data Availability Statement

The raw data supporting the conclusions of this manuscript will be made available by the authors, without undue reservation, to any qualified researcher.

## Ethics Statement

All animal procedures and study protocols were approved by the Experimental Committee of Nanjing Normal University.

## Author Contributions

ML conceived and designed the experiments. JW, GC, QZ, FZ, XY, and XM performed the experiments. ML and JW analyzed the data. ML and JW wrote the manuscript.

## Funding

This study was supported by the National Natural Science Foundation of China (grants 31870895, 31171135), the Priority Academic Program Development of Jiangsu Higher Education Institutions (PAPD), and Top-notch Academic Programs Project of Jiangsu Higher Education Institutions (TAPP).

## Conflict of Interest

The authors declare that the research was conducted in the absence of any commercial or financial relationships that could be construed as a potential conflict of interest.

## Abbreviations

Phil, Phillyrin; RANKL, receptor activator of nuclear factor κB ligand; M-CSF, macrophage colony-stimulating factor; BMMs, bone marrow–derived macrophages; ALP, alkaline phosphatase; CTSK, cathepsin K; TRAP, tartrate resistant acid phosphatase; CTR, calcitonin receptor; ATP6V0d2, ATPase H^+^ transporting V0 subunit d2; MMP9, matrix metallopeptidase 9; NFATc1, nuclear factor of activated T-cells cytoplasmic 1; MAPK, mitogen-activated protein kinase; ERK, extracellular signal-regulated kinase; JNK, c-Jun N-terminal kinases; NF-κB, nuclear factor κB; micro-CT, micro–computed tomography; BMD, bone mineral density; BV/TV, bone volume/tissue volume; OcS/BS, osteoclast surface per bone surface; LPS, lipopolysaccharide.
